# Supplementary material for: High-throughput identification of RNA localization elements in neuronal cells
Source: Nucleic Acids Res. 2022 Sep 15;50(18):10626–42. doi: 10.1093/nar/gkac763 (PMC9561290; doi:10.1093/nar/gkac763)
Supplement: gkac763_Supplemental_Files [file gkac763_supplemental_files.zip › Supplement_NAR_Revision.pdf]

SUPPLEMENTARY FIGURE LEGENDS

Figure S1. (A) Z-normalized neurite localization values for all genes in all samples quantified. The source of each sample is displayed along the x-axis. Neurite-enriched genes chosen for MPRA analysis are indicated with purple dots. Soma-enriched genes chosen for MPRA analysis are indicated in blue dots. The non-enriched gene chosen for MPRA analysis is in black. (B) iPS-derived human motor neurons were fractionated into soma and neurite fractions. Z-normalized neurite enrichments for the RNAs from selected genes are shown. Genes in purple are orthologs of the neurite-enriched mouse genes chosen for MPRA analysis. Genes in blue are orthologs of the soma-enriched mouse genes chosen for MPRA analysis. The gene in black is the ortholog of the non-enriched gene in the MPRA. Wilcoxon p values represent the differences in neurite localization distributions between the indicated genes and all genes (gray). (C) smFISH imaging of Firefly luciferase reporter transcripts fused to the entire 3′ UTRs of the indicated genes.

Figure S2. (A) Ten thousand simulated MPRA oligonucleotides were drawn from mouse chromosome 1 using neighboring oligo step sizes of 2, 5, or 10 nucleotides. A mock fastq containing 10 million reads was then made from these oligonucleotides, including 1 nt deletions and mutations at per-base rates of 0.001 and 0.002, respectively. These reads were then aligned to the mouse genome using bowtie2 using the following parameters:

bowtie2 -q –end-to-end –fr –no-discordant –no-unal -p 4 -x Bowtie2Index/index -1 forreads.fastq -2 revreads.fastq -S sample.sam

The number of reads correctly assigned in each simulated MPRA is shown. (B) Mapping qualities of correctly assigned (gray) and incorrectly assigned (red) reads. (C) Alignment of mock MPRA reads using the same parameters as in (A) with the addition of -D 150. The default value for -D is 15. (D) Number of insertions, deletions, and mutations observed per oligonucleotide in the pool of oligonucleotides. (E) Distribution of error positions across the oligonucleotide. Note that the middle of the oligonucleotide is sequenced by both reads of a paired end sequencing reaction, and errors there were called only if they were present in both reads. The higher rate of errors outside of this middle region therefore likely consists predominantly of sequencing errors. (F) Analysis of the integration efficiency of reporter constructs into cultured cells. Plasmids containing 15mer random sequences were integrated into ~6 million cells of the integrated line. Following selection for integrants, RNA from each of the line was sequenced, and the number of unique 15mer sequences was calculated. To determine the total number of unique 15mers in the cell population, the number of unique sequences was calculated in subsamples of the data. The relationship between the depth of subsampling and the number of unique 15mers was fit to a quadratic polynomial function, and the number of integrants in the cell pool was defined as the maximum of this function. (G) Distribution of oligonucleotide abundances in the integrated GFP reporter transcript in N2A neuronal cells.

Figure S3. (A) Hierarchical clustering of oligonucleotide abundances from the firefly luciferase reporter in CAD cells. (B) Heatmap of oligonucleotide abundances for all significantly enriched (FDR < 0.01) GFP reporters in the CAD samples. Values are Z-normalized in a row wise fashion. (C) Heatmap of oligonucleotide abundances for all significantly enriched (FDR < 0.01) GFP reporters in the N2A samples. Values are Z-normalized in a row wise fashion. (D) Correlation of neurite enrichments between CAD and N2A samples for all GFP reporters. (E) Correlation of neurite enrichments between CAD and N2A samples for all firefly luciferase reporters. (F) Distribution of absolute differences in neurite enrichment between neighboring oligonucleotides. As a control, the positional relationship between all oligonucleotides was randomly shuffled, and the distances between neighboring oligonucleotides were recalculated. (G) Number of significantly neurite- and soma-enriched oligonucleotides among those drawn from UTRs for each gene.

Figure S4. (A) Distribution of neurite enrichment values for oligonucleotides as a function of their position within the 3′ UTR for oligonucleotides taken from the 3′ UTRs of neurite-enriched genes. Data is from the GFP reporter with CAD cell values in pink and N2A values in green. Lines represent a sliding average of 8 oligonucleotides, and the ribbon represents the standard deviation of neurite enrichment for the oligonucleotides in the sliding window. Dots below the lines represent the locations of significantly neurite-localized oligonucleotides (FDR < 0.05). Blue boxes represent the locations of “active windows” defined using the CAD data. Note that the entire 3′ UTRs of *Rab13*, *Akap12*, and *Kif5b* were not sufficient to drive localization of the reporter transcript. (B) As in A, but for genes that were not neurite-enriched. (C) Kmers enriched in neurite-enriched oligonucleotides as defined by cWords (40). (D, E, F) Distribution of A/G content across the 3′ UTRs of *Cdc42bpg* (D), *Rab13* (E), and *Cplx2* (F) and the location of windows of active oligonucleotides, as defined by Figure 4I. (G) The median minimum free energy of oligonucletides defined as soma-, neurite-, or non-enriched as calculated by RNAfold (28). To compare groups, Wilcoxon ranksum tests were performed. (H) The number of guanosine residues participating in G-quadruplexes of oligonucletides defined as soma-, neurite-, or non-enriched as calculated by RNAfold (28). To compare groups, Wilcoxon ranksum tests were performed.

Figure S5. (A) Neurite enrichment of peak oligonucleotide-containing reporter transcripts as assayed by cell fractionation and RT-qPCR. (B) Length of oligonucleotide intersections, as defined by figure 5A. Cplx2 is an outlier in this analysis as the length of the active window was so long such that there was no sequence present in every active oligo. For this gene, then, the length of the oligonucleotide union is shown. (C) Schematic of guide RNA design for the removal of endogenous sequences containing peak oligonucleotides (purple) from endogenous UTRs. Primer designs for the interrogation of CRISPR clones are also shown. For the gel in D, genomic DNA was amplified with primers F1 and R1. For the qPCR-based interrogation of CRISPR clones, the amplification of two amplicons was compared. Amplification using primers Fq2 and Rq2 should occur in all alleles, wildtype or mutated. Conversely, amplification using primers Fq1 and Rq1 should only occur in alleles in which the purple sequence remains and is in the correct orientation (i.e. wildtype alleles). By comparing the ratios of these amplicons using qPCR in wildtype cells and CRISPR clones, we can assess the fraction of wildtype allele

remaining in each clone. (D) Agarose gel showing loss of the sequence in between the two guide RNA cut sites for *Net1* and *Trak2* 3′ UTRs. All significance tests were performed using a t-test. p value notation: * < 0.05, ** < 0.01, *** < 0.001, **** < 0.0001.

Figure S6. (A) RBPs derived from CAD extract that were significantly different in abundance (FDR < 0.05) in the *Net1* peak oligonucleotide RNA pulldown than the control RNA pulldown. (B) As in A, but using N2A cellular lyaste. (C) As in B, but using the *Trak2* peak oligonucleotide as the RNA bait. (D) Western blot of RNA pulldowns from N2A and CAD cell lysate using RNA baits composed of peak oligonucleotides (Net1 and Trak2) or a portion of the coding sequence of firefly luciferase (control). (E) Efficiency of APC, Hnrnpa2, and Unk knockdown as measured by RT-qPCR. (F) Efficiency of Hnrnpa2 knockdown as measured by western blotting. (G) Neurite-enrichments, as determined by cell fractionation and RT-qPCR, of *Net1* and *Trak2* peak oligonucleotide reporter transcripts following the siRNA-mediated knockdown of Hnrnpa2. (H) As in G, but following the knockdown of APC. All significance tests were performed using a Wilcoxon rank-sum test. p value notation: * < 0.05, ** < 0.01, *** < 0.001, **** < 0.0001.

Table S1. Enrichments of all oligonucleotides for the GFP reporter in CAD cells.

Table S2. Enrichments of all oligonucleotides for the Firefly reporter in CAD cells.

Table S3. Enrichments of all oligonucleotides for the GFP reporter in N2A cells.

Table S4. Enrichments of all oligonucleotides for the Firefly reporter in N2A cells.

Table S5. Enrichment of proteins associated with the Net1 peak oligonucleotide RNA as compared to a control RNA sequence in CAD cell extract as determined by mass spectrometry.

Table S6. Enrichment of proteins associated with the Trak2 peak oligonucleotide RNA as compared to a control RNA sequence in CAD cell extract as determined by mass spectrometry.

Table S7. Enrichment of proteins associated with the Net1 peak oligonucleotide RNA as compared to a control RNA sequence in N2A cell extract as determined by mass spectrometry.

Table S8. Enrichment of proteins associated with the Trak2 peak oligonucleotide RNA as compared to a control RNA sequence in N2A cell extract as determined by mass spectrometry.

Supplementary file 1. Sequences for the oligonucleotides used in the MPRA. These sequences do not contain the 20 nt adapters used as PCR handles that were fused to either end of each oligo.

Supplementary file 2. Genomic coordinates for each oligonucleotide member of the MPRA.

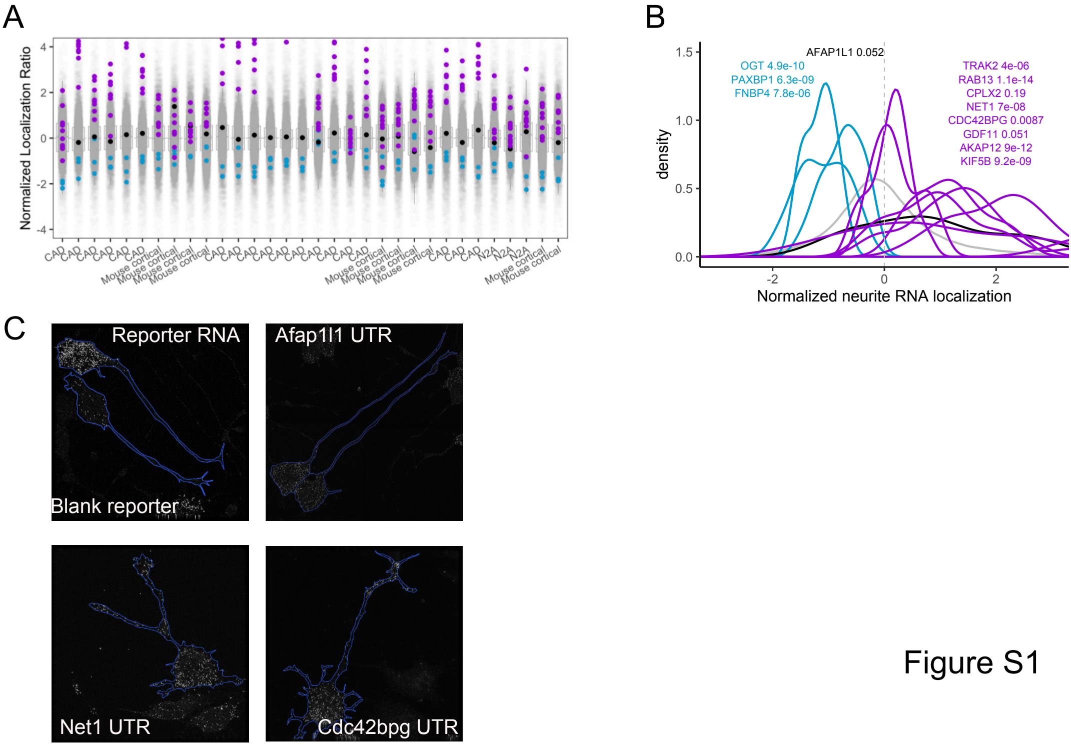
A
Normalized Localization Ratio
B
density
Normalized neurite RNA localization
AFAP1L1 0.052
OGT 4.9e-10
PAXBP1 6.3e-09
FNBP4 7.8e-06
TRAK2 4e-06
RAB13 1.1e-14
CPLX2 0.19
NET1 7e-08
CDC42BPG 0.0087
GDF11 0.051
AKAP12 9e-12
KIF5B 9.2e-09
C
Reporter RNA
Blank reporter
Afap1l1 UTR
Net1 UTR
Cdc42bpg UTR

Figure S1

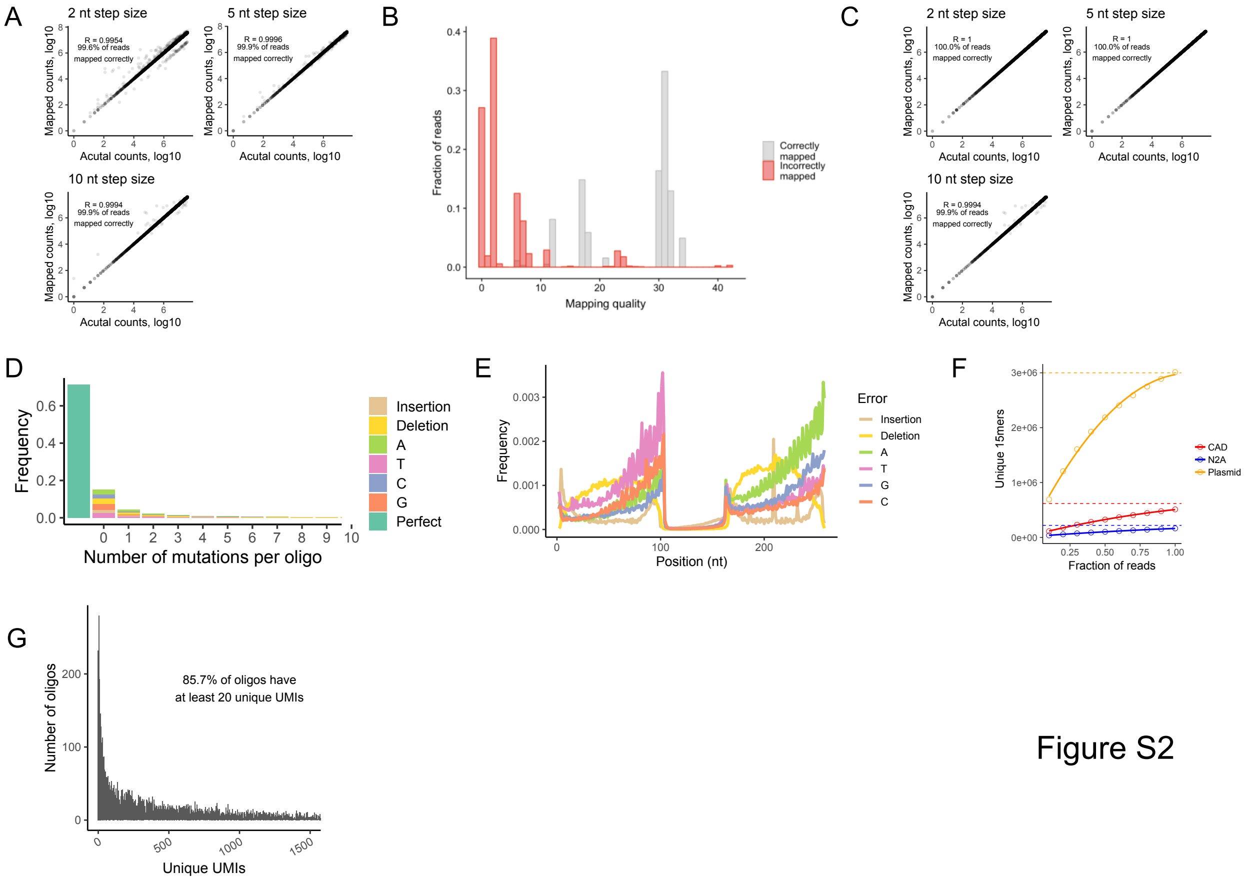

A
2 nt step size
R = 0.9954
99.6% of reads
mapped correctly
5 nt step size
R = 0.9996
99.9% of reads
mapped correctly
10 nt step size
R = 0.9994
99.9% of reads
mapped correctly
Mapped counts, log10
Acutal counts, log10
B
Fraction of reads
Mapping quality
Correctly mapped
Incorrectly mapped
C
2 nt step size
R = 1
100.0% of reads
mapped correctly
5 nt step size
R = 1
100.0% of reads
mapped correctly
10 nt step size
R = 0.9994
99.9% of reads
mapped correctly
D
Frequency
Number of mutations per oligo
Insertion
Deletion
A
T
C
G
Perfect
E
Frequency
Position (nt)
Error
Insertion
Deletion
A
T
G
C
F
Unique 15mers
Fraction of reads
CAD
N2A
Plasmid
G
Number of oligos
85.7% of oligos have
at least 20 unique UMIs
Unique UMIs

Figure S2

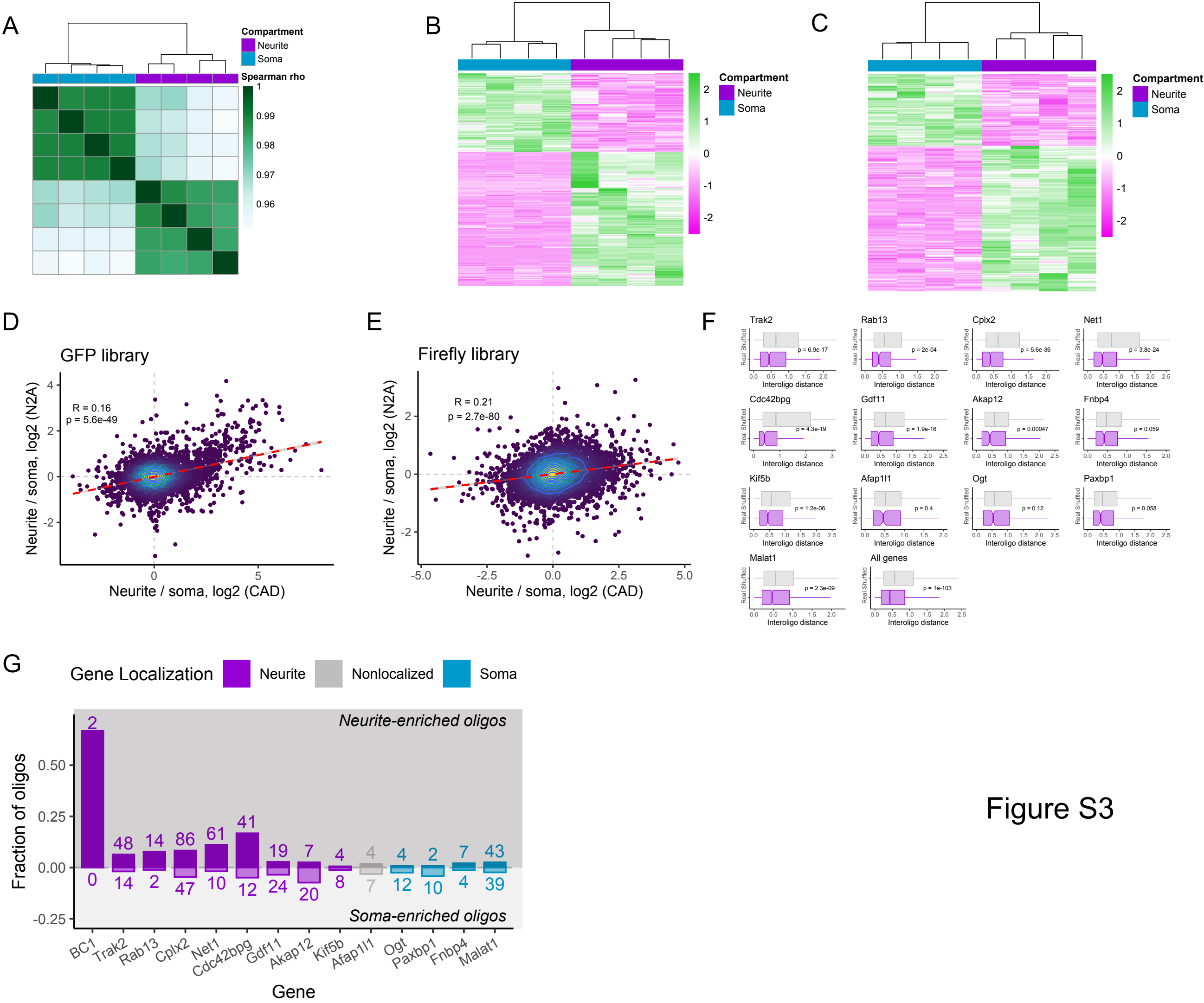

A
Compartment
Neurite
Soma
Spearman rho
1
0.99
0.98
0.97
0.96
B
C
Compartment
Neurite
Soma
2
1
0
-1
-2
D
GFP library
R = 0.16
p = 5.6e-49
Neurite / soma, log2 (N2A)
Neurite / soma, log2 (CAD)
E
Firefly library
R = 0.21
p = 2.7e-80
Neurite / soma, log2 (N2A)
Neurite / soma, log2 (CAD)
F
Trak2
p = 6.9e-17
Rab13
p = 2e-04
Cplx2
p = 5.6e-36
Net1
p = 3.8e-24
Cdc42bpg
p = 4.3e-19
Gdf11
p = 1.9e-16
Akap12
p = 0.00047
Fnbp4
p = 0.059
Kif5b
p = 1.2e-06
Afap1l1
p = 0.4
Ogt
p = 0.12
Paxbp1
p = 0.058
Malat1
p = 2.3e-09
All genes
p = 1e-103
Real Shuffled
Interoligo distance
G
Gene Localization
Neurite
Nonlocalized
Soma
Neurite-enriched oligos
Soma-enriched oligos
Fraction of oligos
Gene
BC1
Trak2
Rab13
Cplx2
Net1
Cdc42bpg
Gdf11
Akap12
Kif5b
Afap1l1
Ogt
Paxbp1
Fnbp4
Malat1

Figure S3

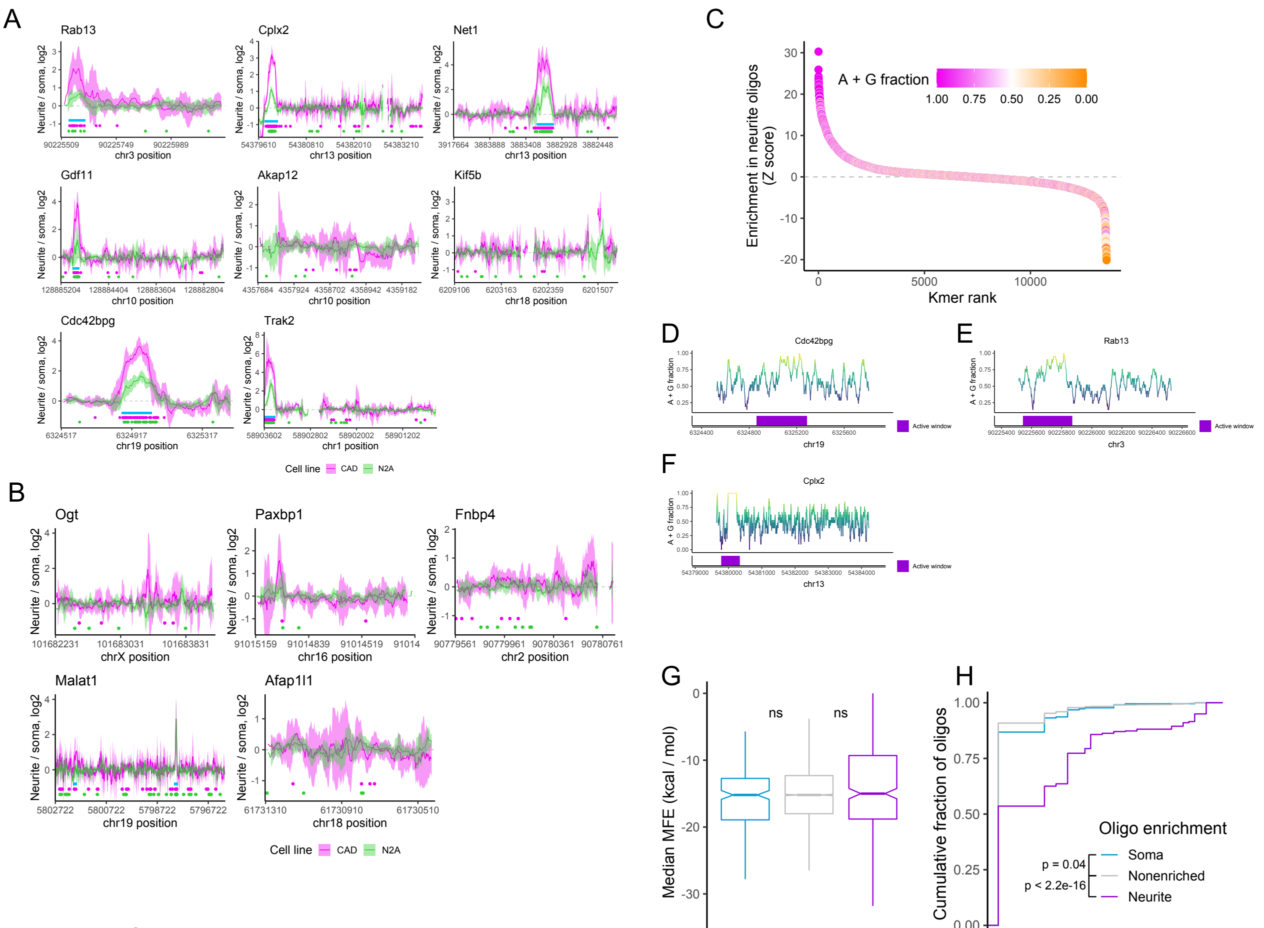

A
Rab13
Cplx2
Net1
Gdf11
Akap12
Kif5b
Cdc42bpg
Trak2
Neurite / soma, log2
chr3 position
chr13 position
chr10 position
chr18 position
chr19 position
chr1 position
Cell line
CAD
N2A
B
Ogt
Paxbp1
Fnbp4
Malat1
Afap1l1
chrX position
chr16 position
chr2 position
chr19 position
chr18 position
C
A + G fraction
1.00 0.75 0.50 0.25 0.00
Enrichment in neurite oligos (Z score)
Kmer rank
D
Cdc42bpg
E
Rab13
F
Cplx2
A + G fraction
Active window
chr19
chr3
chr13
G
Median MFE (kcal / mol)
ns
ns
Soma
Nonenriched
Neurite
Oligo enrichment
H
Cumulative fraction of oligos
Oligo enrichment
p = 0.04
p < 2.2e-16
Soma
Nonenriched
Neurite
Number of Gs in quadruplex

Figure S4

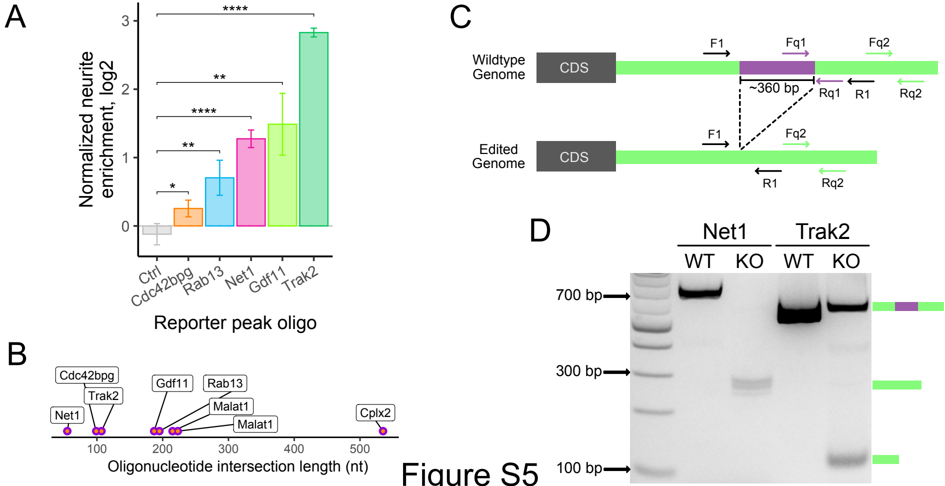

A
Normalized neurite enrichment, log2
0
1
2
3
****
**
****
**
*
Ctrl
Cdc42bpg
Rab13
Net1
Gdf11
Trak2
Reporter peak oligo
B
Cdc42bpg
Trak2
Net1
Gdf11
Rab13
Malat1
Malat1
Cplx2
100
200
300
400
500
Oligonucleotide intersection length (nt)
C
Wildtype Genome
CDS
F1
Fq1
Fq2
~360 bp
Rq1
R1
Rq2
Edited Genome
CDS
F1
Fq2
R1
Rq2
D
Net1
Trak2
WT
KO
WT
KO
700 bp
300 bp
100 bp

Figure S5

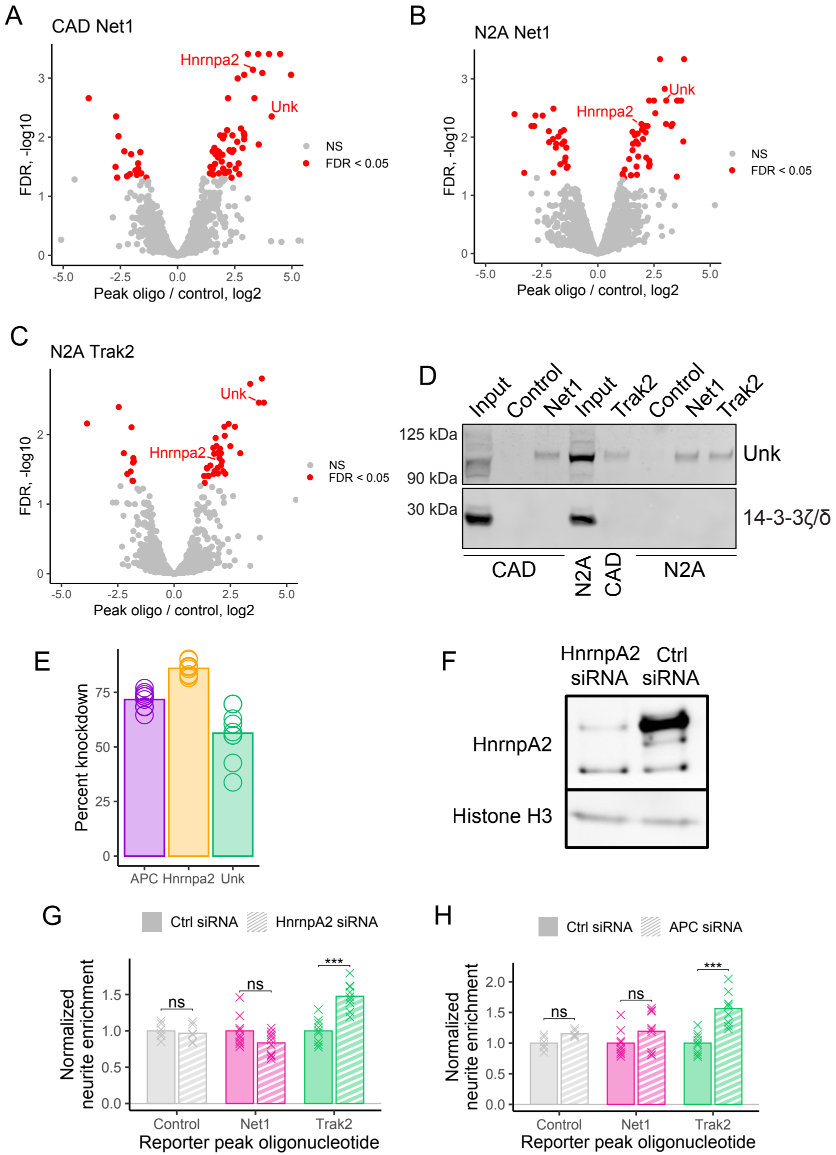
A
CAD Net1
Hnrnpa2
Unk
NS
FDR < 0.05
FDR, -log10
Peak oligo / control, log2
B
N2A Net1
Unk
Hnrnpa2
NS
FDR < 0.05
FDR, -log10
Peak oligo / control, log2
C
N2A Trak2
Unk
Hnrnpa2
NS
FDR < 0.05
FDR, -log10
Peak oligo / control, log2
D
Input
Control
Net1
Input
Trak2
Control
Net1
Trak2
125 kDa
90 kDa
30 kDa
Unk
14-3-3ζ/δ
CAD
N2A
CAD
N2A
E
Percent knockdown
APC
Hnrnpa2
Unk
F
HnrnpA2 siRNA
Ctrl siRNA
HnrnpA2
Histone H3
G
Ctrl siRNA
HnrnpA2 siRNA
Normalized neurite enrichment
ns
ns
***
Control
Net1
Trak2
Reporter peak oligonucleotide
H
Ctrl siRNA
APC siRNA
Normalized neurite enrichment
ns
ns
***
Control
Net1
Trak2
Reporter peak oligonucleotide

Figure S6
